# P-717. Serologic response to doxycycline versus penicillin for treatment of early or late stage syphilis

**DOI:** 10.1093/ofid/ofaf695.929

**Published:** 2026-01-11

**Authors:** Catherine A Packard, Emily Kirkpatrick, Amber Welborn, Rose Allen, Travis J Carlson

**Affiliations:** University Hospital, Columbia, SC; University Health, San Antonio, Texas; University Health, San Antonio, Texas; University Health, San Antonio, Texas; The University of Texas at Austin College of Pharmacy, San Antonio, Texas

## Abstract

**Background:**

With intermittent barriers to penicillin use for syphilis treatment, alternative treatment strategies are needed. Some literature supports doxycycline, but data are limited in certain subgroups, such as people living with human immunodeficiency virus (PLWHIV) and those with late stages of syphilis.

**Methods:**

This was a single-center, retrospective cohort study that included adults with a positive syphilis screen between 7/1/2020-11/30/2024 who received either doxycycline or penicillin. Patients were excluded if they received suboptimal treatment, were pregnant or incarcerated, or did not have a follow-up rapid plasma reagent (RPR). The primary outcome was serologic response, defined as a four-fold or greater decrease in RPR from baseline to 12 months. Patients were stratified by antibiotic treatment and matched 1:1 by age, sex, race, HIV status, baseline RPR, and stage of syphilis using nearest-neighbor matching without replacement. All p-values were two-sided, and p < 0.05 was considered statistically significant.

**Results:**

A total of 110 patients were included: 80 in the penicillin group and 30 in the doxycycline group. The majority of patients were male (77%), non-white (75%), and PLWHIV (70%) with a mean (standard deviation [SD]) age of 38 (±10) years. In the penicillin group, 58/80 (73%) patients achieved serologic response versus 24/30 (80%) patients in the doxycycline group (p = 0.42). The mean (SD) time to serologic response was similar between groups: 165 (±69) days in the penicillin group versus 183 (±79) days in the doxycycline group. After matching, 60 patients remained: 30 in each group. In the penicillin group, 23/30 (77%) patients achieved serologic response versus 24/30 (80%) patients in the doxycycline group (p = 0.75). The mean (SD) time to serologic response was similar between groups: 163 (±73) versus 183 (±79) days in the penicillin and doxycycline groups, respectively.

**Conclusion:**

Patients with syphilis who were treated with doxycycline had a similar rate of serologic response when compared to patients treated with penicillin. These findings suggest that doxycycline can be utilized as an alternative therapy in many patients with syphilis, regardless of stage or HIV status.

**Disclosures:**

Travis J. Carlson, PharmD, BCIDP, Aimmune Therapeutics, Inc.: Speaker bureau

